# Cucumber Seedling Segmentation Network Based on a Multiview Geometric Graph Encoder from 3D Point Clouds

**DOI:** 10.34133/plantphenomics.0254

**Published:** 2024-10-16

**Authors:** Yonglong Zhang, Yaling Xie, Jialuo Zhou, Xiangying Xu, Minmin Miao

**Affiliations:** ^1^ College of Information Engineering (College of Artificial Intelligence), Yangzhou University, Yangzhou, Jiangsu 225127, China.; ^2^ College of Horticulture and Landscape Architecture, Yangzhou University, Yangzhou, Jiangsu 225009, China.

## Abstract

Plant phenotyping plays a pivotal role in observing and comprehending the growth and development of plants. In phenotyping, plant organ segmentation based on 3D point clouds has garnered increasing attention in recent years. However, using only the geometric relationship features of Euclidean space still cannot accurately segment and measure plants. To this end, we mine more geometric features and propose a segmentation network based on a multiview geometric graph encoder, called SN-MGGE. First, we construct a point cloud acquisition platform to obtain the cucumber seedling point cloud dataset, and employ CloudCompare software to annotate the point cloud data. The GGE module is then designed to generate the point features, including the geometric relationships and geometric shape structure, via a graph encoder over the Euclidean and hyperbolic spaces. Finally, the semantic segmentation results are obtained via a downsampling operation and multilayer perceptron. Extensive experiments on a cucumber seedling dataset clearly show that our proposed SN-MGGE network outperforms several mainstream segmentation networks (e.g., PointNet++, AGConv, and PointMLP), achieving mIoU and OA values of 94.90% and 97.43%, respectively. On the basis of the segmentation results, 4 phenotypic parameters (i.e., plant height, leaf length, leaf width, and leaf area) are extracted through the K-means clustering method; these parameters are very close to the ground truth, and the *R*^2^ values reach 0.98, 0.96, 0.97, and 0.97, respectively. Furthermore, an ablation study and a generalization experiment also show that the SN-MGGE network is robust and extensive.

## Introduction

Phenotypic analysis of plant morphological traits, including plant height, leaf attributes, fruit characteristics, and floral features, holds paramount importance in plant breeding endeavors and precision agriculture management. This analysis provides a vital assessment of plant growth development, tolerance, and yield. Traditional plant phenotyping approaches rely heavily on manual measurement, have low efficiency and large errors, are labor intensive, and are unable to adapt to high-throughput plant phenotype acquisitions. In recent years, rapid advancements in computer vision and sensing technologies have substantially facilitated the acquisition and analysis of high-throughput and high-quality phenotyping data, e.g., 3-dimensional (3D) data. Various tools and techniques, such as depth cameras [1], time-of-flight cameras [2], light detection and ranging (LiDAR) [3], and multiview stereo (MVS) reconstruction [4–6], have been widely used to collect 3D plant data and extract various phenotypic parameters, such as plant height, leaf area, leaf length, width, and leaf inclination. As a 3D reconstruction technology from multiple images, MVS is cost-effective and boasts high accuracy and robustness against occlusion and overlapping between plants. Wu et al. [4] devised MVS-Pheno, a portable, cost-effective platform tailored for high-throughput phenotypic analysis of individual plants in the field. Sun et al. [7] further expanded the horizons by reconstructing 3D images of 5 distinct soybean varieties across their entire growth cycle, spanning 13 developmental stages. Saeed et al. [8] introduced a framework for the 3D reconstruction of peanut plants, named PeanutNeRF. This approach integrates 2D and 3D data and employs NeRF [6] to train 3D radiance fields, obtaining an implicit representation of the plants. Hu et al. [9] built a real plant phenotypic dataset on which they investigated the advantages and limitations of several state-of-the-art NeRF models.

Unlike 2D images, 3D data incorporate depth and spatial information in addition to color and texture information. It can achieve more precise recognition of boundaries and intricate details, extracting local features with higher accuracy. Recent 3D-based deep learning methods for plant segmentation and phenotype extraction have garnered considerable attention from researchers in the field. For 3D point cloud segmentation tasks, Qi et al. [10] innovatively proposed PointNet, an end-to-end deep learning segmentation network designed to directly process unordered point clouds, which offered the capability of object classification and pointwise semantic segmentation. They subsequently leveraged PointNet within the hierarchical structures to capture the local geometric information (PointNet++ [11]). Thomas et al. [12] proposed a deformable convolution with kernel points, named KPConv, to learn local geometry. Owing to the powerful ability of graph convolution, some researchers treat points as nodes of a graph to extract local geometric features from local points. For example, Wang et al. [13] constructed a k-nearest neighbor (KNN) graph for each point within the feature space and subsequently utilized EdgeConv operators to extract local geometric features. Lin et al. [14] proposed a graph convolution network with deformable 3D kernels, termed 3D-GCN, which maintains shift and scale invariance properties in the point clouds. Wei et al. [15] designed adaptive kernels for points according to their dynamically learned features. In the field of plant point cloud segmentation, a variety of methods for distinct plant species have been proposed [16–23]. Li et al. designed 2 organ-level segmentation networks, PlantNet [16] and PSegNet [17], on a point cloud dataset of 3 species, i.e., tobacco, tomato, and sorghum. The average intersection over union (IoU) of semantic segmentation reached 85.86% on PlantNet and 89.90% on PSegNet. Guo et al. [24] proposed a cabbage point cloud segmentation model that combines PointNet++ and an attention module, which achieves an 86% IoU. Similarly, Yang et al. [22] leveraged the PointNet++ architecture as their semantic segmentation model with a 91.93% mIoU, as well as the hierarchical aggregation approach, for instance, segmentation with an 89.57% mean average precision (mAP) on a maize point cloud dataset. Du et al. [20] proposed a plant segmentation transformer (PST) for rapeseed point clouds acquired via handheld laser scanning (HLS), which comprises a dynamic voxel feature encoder coupled with dual window set attention blocks. The network achieved superior performance in semantic and instance segmentation tasks. Yun et al. [23] provided a comprehensive overview of various deep learning methods that have been applied in the field of forest studies, including the extraction of forest phenotypic parameters from point clouds.

The aforementioned methodologies for handling point clouds with non-Euclidean spatial configurations focus on embedding them into Euclidean spaces, simultaneously encoding both the topological and semantic information. Indeed, 3D objects represented as point clouds inherently embody a compositional nature, wherein simpler components are intricately assembled to progressively form more elaborate shapes. This nature results in an implicit hierarchy, such as a tree-like structure. However, Euclidean measures are inadequate for providing the most powerful or meaningful part-whole geometrical representations for structured data in a non-Euclidean space. In contrast, hyperbolic space with constant negative curvature is more suitable for capturing the nonlinear growth rate of hierarchical structures while minimizing distortion and producing high-quality representations, even within low-dimensional embedding spaces [25–28]. Recently, hyperbolic space has garnered significant popularity in processing point cloud data. Montanaro et al. [29] embedded point cloud features from Euclidean space into hyperbolic space and introduced a regularizer to effectively promote the part-whole hierarchy. Cheng et al. [30] employed a dynamic hyperbolic graph convolution module with learnable curvature to better represent the local geometric topology within the point clouds. Furthermore, a reweighting strategy was used to adjust the importance of the original features to optimize the feature descriptor.

To date, the study of plant phenotype analysis from hyperbolic space is still unexplored. This study proposed a novel semantic segmentation network, called SN-MGGE, on constructed cucumber seedling point clouds through multiple views, such as Euclidean space and hyperbolic space. The contributions of our current study are 3-fold: (a) Well-labeled high-quality 3D point clouds of individual cucumber seedling plants based on VisualSFM reconstruction are constructed. (b) A geometric graph encoder (GGE) is introduced to capture both local geometric features and hierarchical structure information across both Euclidean and hyperbolic spaces simultaneously, which enhances the understanding and representation of complex geometric data by leveraging the advantages of both Euclidean and hyperbolic geometries. Additionally, a semantic segmentation network (SN-MGGE) is designed to achieve automatic point cloud segmentation of cucumber seedlings from individual plants to organs. Finally, phenotype parameters are extracted from the segmented cucumber seedling point clouds via the clustering method. (c) The segmentation efficiency and generalization ability of SN-MGGE are verified on the point clouds of cucumber seedlings and the other 3 plant species. The test results indicate that SN-MGGE performs well in processing point cloud data from different plant species.

## Materials and Methods

### Overview

**Fig. 1.**
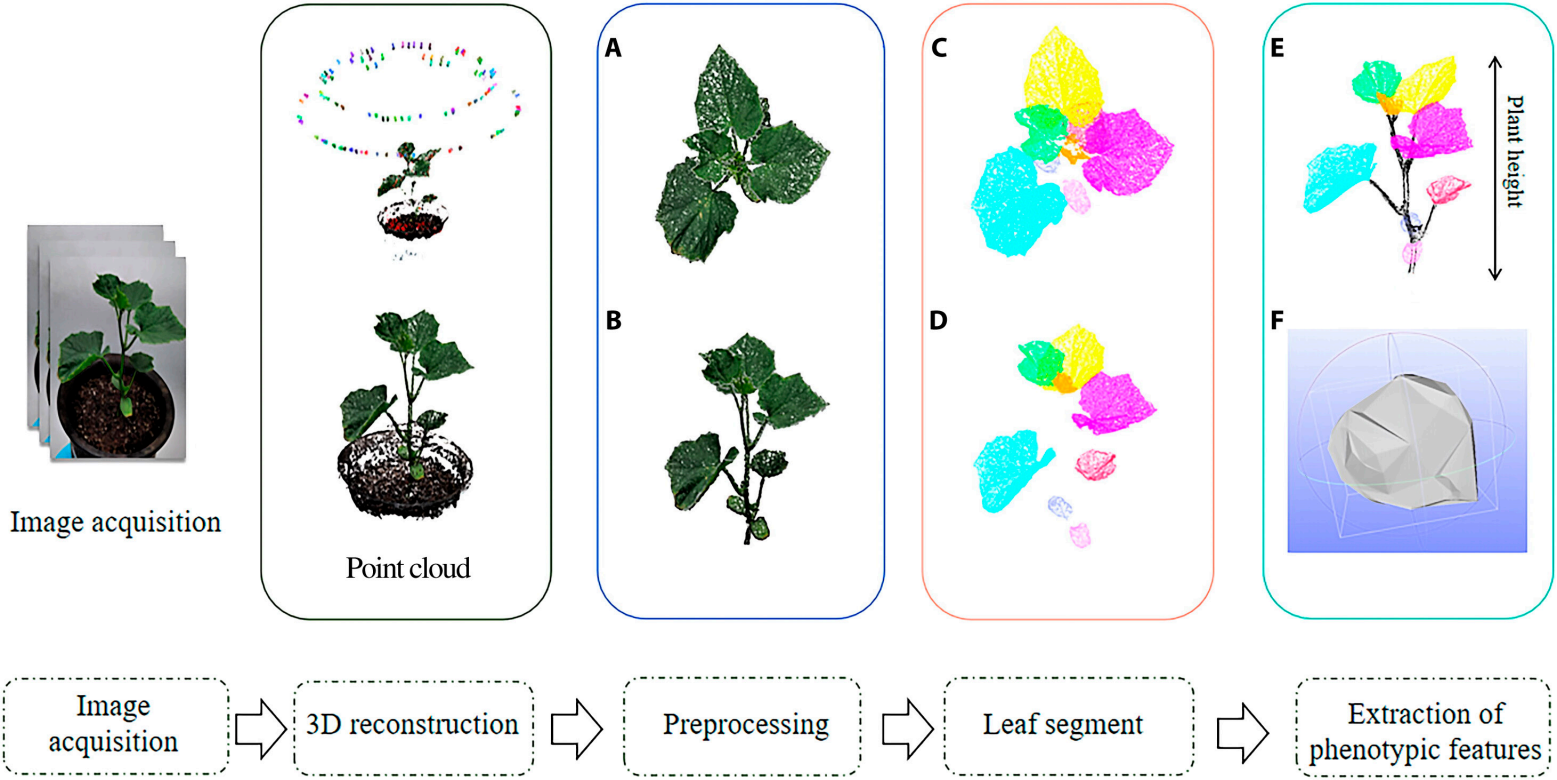
Overview of the proposed method. (A) Top view. (B) Side view. (C) Top view. (D) Side view. (E) Plant height. (F) Calculations of leaf length, width, and leaf area.

Figure 1 illustrates the overall process of the proposed method, which comprises 5 stages: image acquisition, 3D point cloud reconstruction, data preprocessing, leaf segmentation, and leaf phenotypic feature extraction.

### Data acquisition

**Fig. 2.**
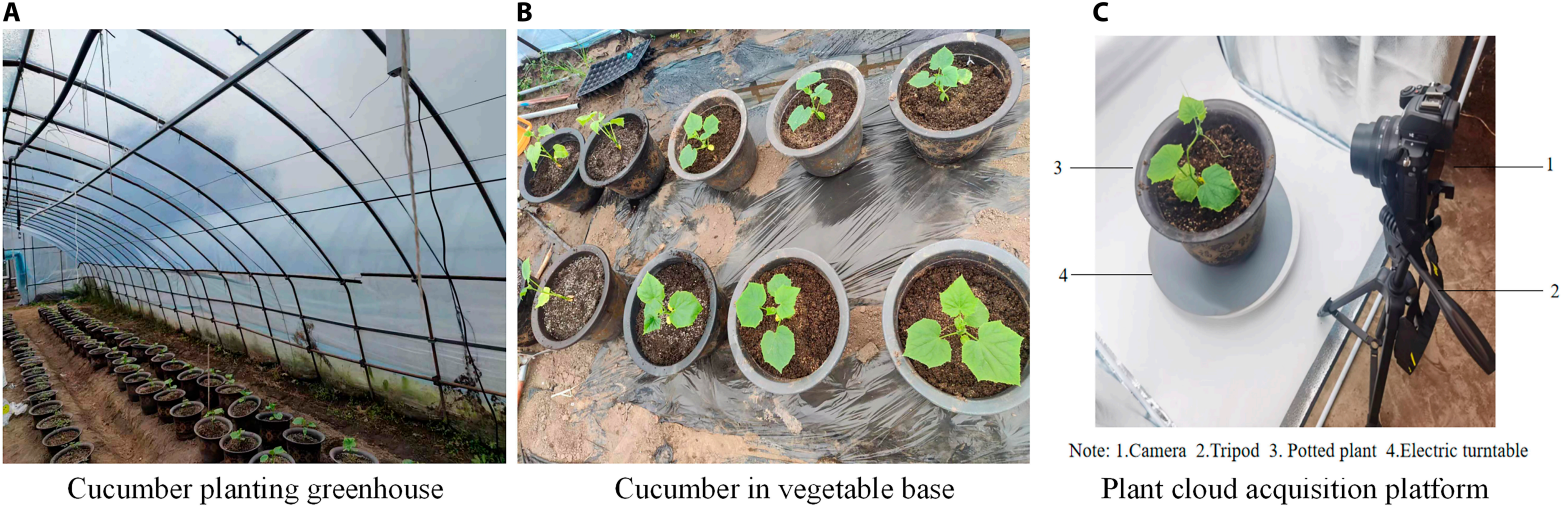
(A to C) Cucumber planting greenhouse and data collection platform.

We chose the high-yielding and disease-resistant cucumber variety Jinchun No. 5 as the primary experimental subject for our study (Fig. 2A and B). From 2023 April 5 to 2023 June 10, 100 diverse cucumber seedlings in the greenhouse of Yangzhou University’s vegetable base (32.29°N, 119.48°E) were used to acquire images for modeling. Seeds were soaked in warm water, followed by germination at a constant temperature of 28 °C in an incubator until 90% of the seeds showed signs of whitening. They were subsequently sown into 50-cell trays with a substrate consisting of a mixture of peat, vermiculite, and perlite at a volumetric ratio of 3:1:1. After sowing, the trays were placed on greenhouse seedbeds with a day/night temperature of 28 °C/18 °C and a relative humidity of 65% to 85%. The plants were transplanted into pots at the 2-leaf stage. Image data collection was performed on the same cucumber seedling every 3 d.

To obtain clear and high-quality images from a better perspective, we built a point cloud acquisition platform composed of a tripod, camera, an electric turntable with a diameter of 42 cm, small photography studio, and computer (Fig. 2C). In the small photography studio, a white backdrop cloth and 2 light-emitting diode (LED) lights were used, providing a solid color shooting background and ample lighting. Furthermore, the camera was mounted on a tripod and precisely positioned to directly face the cucumber seedling while maintaining a fixed horizontal distance of 0.7 m from the center of the turntable. The rotation angle of the turntable was meticulously controlled via remote control, with intermittent pausing every 30° to capture images. This systematic approach enabled the capture of multiview images in a complete 360° rotation around the cucumber seedling. During each collection, an individual cucumber seedling was photographed for 3 to 5 min, capturing approximately 200 high-resolution images from 12 different angles. The phenotypic parameters (including plant height, leaf length, leaf width, and leaf area) of the cucumber seedlings were manually measured after shooting to evaluate the segmentation performance of the SN-MGGE network on the cucumber seedling point cloud dataset.

### 3D point cloud reconstruction

**Fig. 3.**
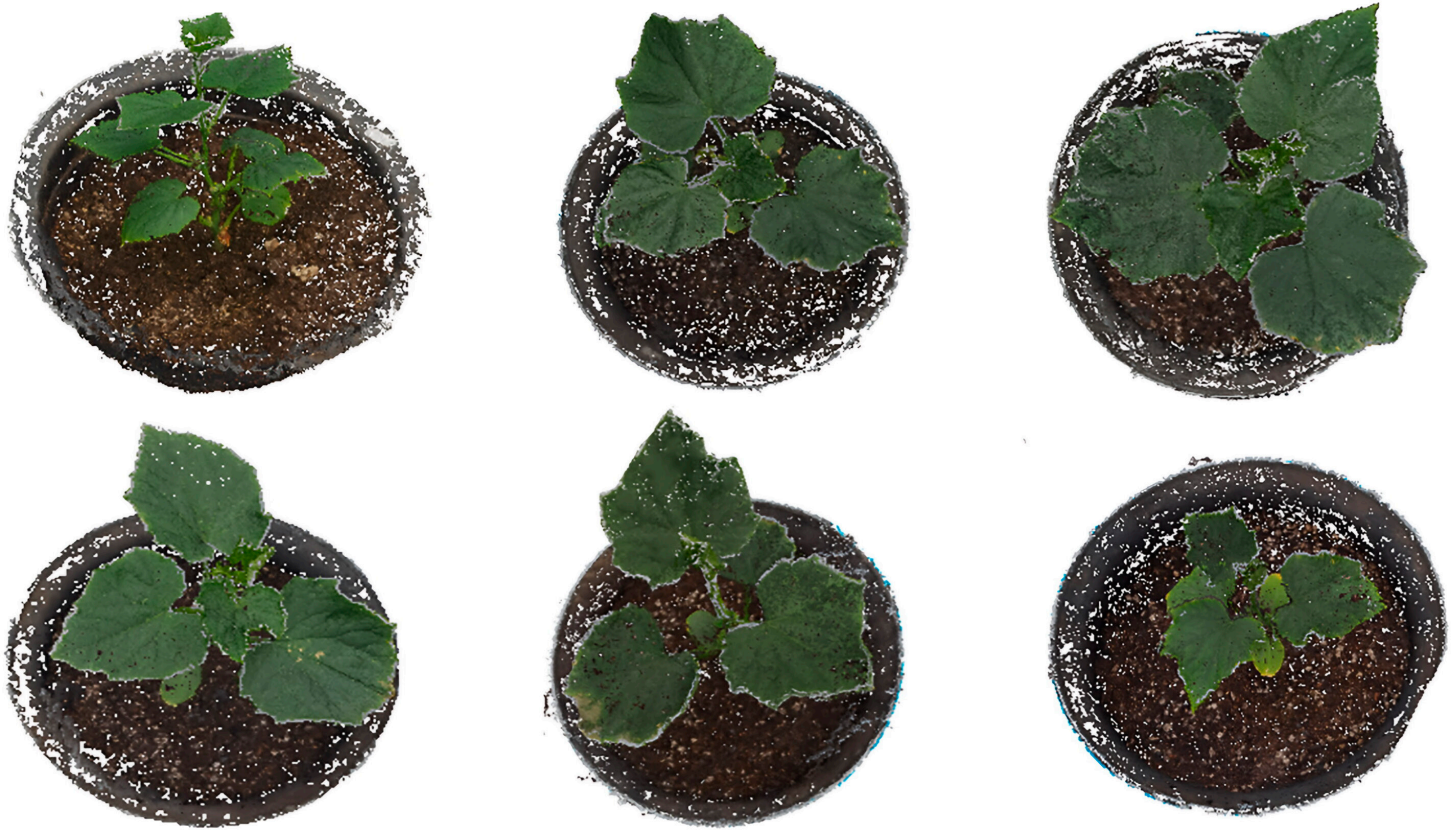
Sample images of reconstructed cucumber seedling point clouds.

We used VisualSFM [31], which is an open-source tool for inferring the 3D structure of objects through analysis of the geometric relationships between images, to generate point clouds of the cucumber seedlings. VisualSFM uses the structure from motion (SfM) [32] algorithm as the core method and involves a number of comprehensive workflow processes: (a) employ the scale-invariant feature transform (SIFT) [33] descriptor to detect and match feature points and the random sample consensus (RANSAC) [34] to filter out mismatched pairs, (b) solve for camera parameters and 3D point coordinates via the SfM method to generate sparse 3D point clouds, and (c) apply the clustering views for multiview stereo (CMVS) and patch-based multiview stereo (PMVS) [35] to produce dense and texture-rich point clouds on the above sparse model. Several image examples of the image sequence in the same coordinate system as the plant model captured by VisualSFM are shown in Fig. 3.

The software can handle a large number of input images; the more images there are, the more detailed the constructed point clouds become. In addition, it can export widely used 3D data formats, such as ply and pcd, for subsequent processing and analysis.

### Data preprocessing

The reconstructed cucumber seedling point clouds inevitably contain some noise points (e.g., outliers and background points), which severely affect the performance of the leaf segmentation network. The noise points are mainly distributed around the surface of the leaf because of the error caused by reflection and mutual occlusion on the leaf surface during feature point pairing. To handle noise points, we employ radius outlier removal (ROR) [36] to calculate the number of points within a specific range around each point and remove those with a count below the threshold.

Furthermore, there are approximately 10,000 to 12,000 points per plant in cucumber seedling point clouds, which is still complicated. Therefore, we adopted a uniform downsampling method to regulate the quantity of points to a range of 4,096, mitigating data complexity while preserving the essential characteristics and structural information of the point clouds. Then, the point clouds were normalized and scaled to the range of [−1, 1] for the *xyz* coordinate values, and the normalization transformation matrix was recorded. Considering the impact of the above 2 operations on the accuracy of subsequent phenotype feature extraction, point clouds are restored to their true size via matrix inversion and upsampling for subsequent calculation.

Finally, we used CloudCompare software [37] to annotate the dataset for leaf segmentation. CloudCompare can visualize, edit, and process large-scale 3D point clouds. During the annotation process, cucumber seedling leaves and stems were manually selected and categorized as 0 for leaves and 1 for stems. In accordance with the ShapeNet Part dataset [38], the point clouds were stored as text files in which each line represents the (*x*,*y*,*z*) coordinates and label number of an individual point.

### Segmentation network

#### Overview

**Fig. 4.**
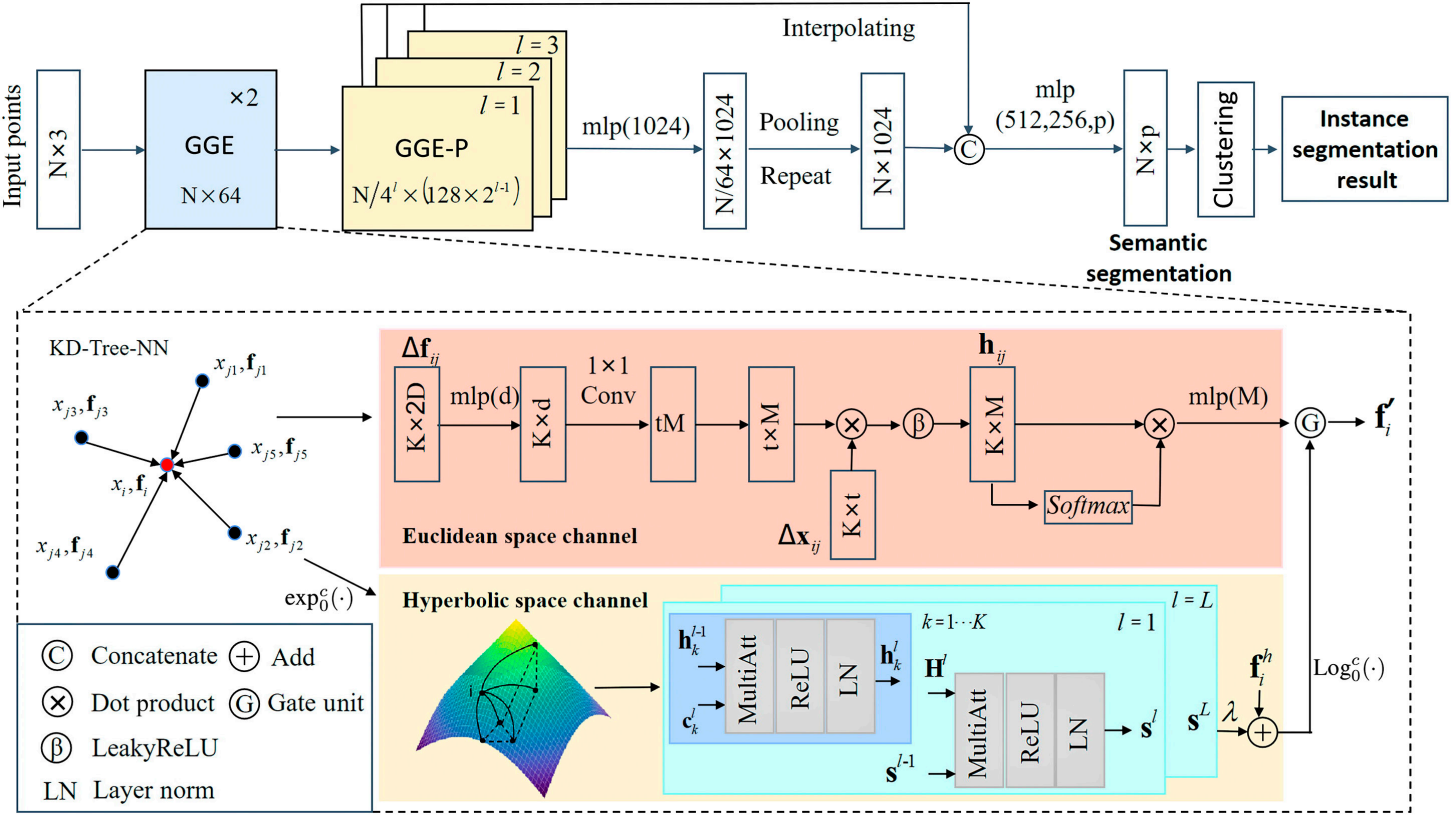
Architecture of SN-MGGE for segmentation. Given the input point clouds, the GGE module progressively extracts local geometric features, as well as geometric structure features, across both the Euclidean and hyperbolic spaces. We reduce the number of points progressively and form a hierarchical structure to obtain diverse granularity features by stacking multiple GGE-P modules. The definitive point features are derived through interpolation and repetition, which are then fed into the MLP layer to generate the segmentation results. Note that *N* is the number of point clouds, *K* is the number of neighbors of the target point, *l* denotes the *l*th layer, λ is a scale factor, and *D*, *d*, *t*, and *M* denote the dimensions. For a target point *x_i_*, the spatial and feature inputs are Δ*x_ij_* = [*x_i_*; *x_j_* − *x_i_*] and Δ*f_ij_* = [*f_i_*; *f_j_* − *f_i_*], respectively.

The overall architecture of the proposed SN-MGGE network is illustrated in Fig. 4 and consists of 2 main components. The front part incorporates 2 consecutive geometric graph encoder (GGE) modules (highlighted in the dashed box in Fig. 4) and 3 consecutive GGE-P modules. The GGE module captures point features by leveraging the local geometric characteristics and hierarchical structure information from point clouds, using the proposed graph encoders over both the Euclidean and hyperbolic spaces. The GGE-P module is a downsampling-like structure in which the number of points is progressively reduced, which combines the GGE module and graph pooling layer to obtain diverse granularity features. In the final part of the network, the above subsampled features are reshaped to the same dimension through interpolating and repeating operations and concatenated for the final point features. We subsequently use the shared multilayer perceptron (MLP) with the number of layer neurons defined as (512, 256, *p*) to yield semantic segmentation results, where *p* is the number of semantic labels. The K-means clustering method [39] is used for instance segmentation, and on the basis of the instance segmentation results, phenotype parameters are extracted.

#### Geometric graph encoder module

Graph construction: Let **X** = {*x*_1_, *x*_2_, . . . , *x_N_*} ∈ **R**^*N*×3^ as the input point clouds, where *N* indicates the number of points and *x_i_* represents the (*x*,*y*,*z*) coordinate position of the *i*th point in Euclidean space. The corresponding features are defined as **F** = {**f***_i_*|*i* = 1, 2, … , *N*} ∈ **R**^*N*×*D*^, where **f***_i_* is the *D*-dimensional feature of the *i*th point, which contains side information (e.g., color and normal) apart from the coordinate value. To capture the local structures, we construct a directed graph ***G****_i_* = (**V***_i_*, **E***_i_*) for each target point **x***_i_* ∈ **X** by employing the kd-tree nearest neighbor algorithm (KD-Tree-NN) [40], where **V***_i_* ⊆ **X** is the set of points (nodes) and where **E***_i_* ⊆ **V***_i_* × **V***_i_* represents the set of edges. Let **N**(*i*) = {*x_j_*|(*x_i_*, *x_j_*) ∈ **E***_i_*} be the neighbor set of point *x_i_*, where *k* = |**N**(*i*)| is the number of neighbors. Next, we take point *x_i_* as an example to illustrate the feature extraction process in detail.

Euclidean space channel: To capture distinctive geometric relationship representations between each pair of points (*x_i_*, *x_j_*) ∈ **E***_i_*, we adopt adaptive convolution to extract point features similar to AGConv [15], which is based on the constructed graph **G***_i_*. First, we use the position encoding (PE) operation to obtain the encoded low-level neighborhood feature set **R***_i_* = {Δ*x_ij_*|*j* = 1, 2, … , *K*} ∈ R^*K*×6^, where Δ**x***_ij_* is defined as [*x_i_*; *x_j_* – *x_i_*], [⋅; ⋅] represents the concatenation operation, and *K* is the number of neighbor points. Similarly, the high-level neighborhood feature set **H***_i_* is defined as {**h***_ij_*|*j* = 1, 2, … , *K*} ∈ **R**^*K*×*M*^, where **h***_ij_* is calculated as follows:

$$h_{\mathrm{ij}}=[\sigma (\langle \Delta x_{\mathrm{ij}}g_{m}(\Delta f_{\mathrm{ij}})\rangle )|m=1,2,\ldots \;,\;M]$$ (1)

where Δ**f***_ij_* is defined as [**f***_i_*; **f***_j_* − **f***_i_*], 〈⋅, ⋅〉 represents the inner-product operation, and *σ* is the activation function LeakyReLU, *g_m_* : R^2*D*^ → R^6^ is the feature mapping function; here, we use a shared MLP.

The final feature $f_{i}^{\;e}$ of the central point *x_i_* is obtained by aggregating the features **h***_ik_* and ∀*k* ∈ [1 … *K*] of all the neighbor points through the attention pooling operation, which is represented by the following operation.

fie=MLP∑k=1Kwhikik (2)

$$w_{i}=\text{softmax}\left(\left[h_{i1};h_{i2};\ldots ;h_{\mathrm{iK}}\right]\right)$$ (3)

where *w_i_* = {*w*_*i*1_, *w*_*i*2_, … , *w_iK_*} represents the weight set.

Hyperbolic space channel: We project the graph structure **G***_i_* of the central point *x_i_* into hyperbolic space to extract the geometric shape structure features. For each point *x_j_* ∈ **V***_i_* ∪ {*x_i_*},

the projected feature vector $f_{j}^{h}$ is defined as [41]:

$$f_{j}^{h}=\exp_{O}^{c}\left(\;f_{j}\;\right)=\text{tahn}\left(\;\sqrt{\left|c\right|}\|f_{j}\|\;\right)\frac{f_{j}}{\sqrt{\left|c\right|}\|f_{j}\|}$$ (4)

where *c* is a negative curvature and ‖⋅‖ denotes the Euclidean norm.

We convert the graph structure **G***_i_* to the star graph $G_{i}^{s}$ through connecting neighbor nodes with a certain probability, i.e., $G_{i}^{s}\;=\;\left(\;V_{i},\;E_{i},\;U\left\{{\left(x_{j},\;x_{h}\right)}_{\mathrm{Pjh}}\left|x_{j},\;x_{h}\in V_{i}\wedge \;j,\;h\neq i,\;0\leq p_{\mathrm{jh}}\leq 1\right.\right\}\;\right)$. On the basis of $G_{i}^{s}$, we propose the hyperbolic geometric structure (HGS) encoder, which is mainly based on the Star Transformer [42], to capture the geometric shape structure features. First, let $H^{0}=\left[f_{1}^{h},\ldots ,f_{k}^{h}\right]$, where $f_{k}^{h}$ is the feature of the *k*th neighbor node *x_k_* ∈ **N**(*i*). The central point x_1_ is called the planet node. Then, we stack *L* layers to update **H***^L^* and *s^L^*; here, $s^{0}=f_{i}^{h}$. At the *l*th layer, for each neighbor node *x_k_*, $h_{k}^{l}$ is updated through the multihead attention mechanism as follows:

$$c_{k}^{l}=\left[h_{k-1}^{i-1};\;h_{k}^{l-1};\;h_{k+1}^{l-1};\;s^{l-1}\right]$$ (5)

$$h_{k}^{l}=\mathrm{LN}\left(\text{RELU}\left(\text{MultiAtt}\left(h_{k}^{l-1},\;c_{k}^{l}\;\right)\;\right)\;\right)$$ (6)

where $c_{k}^{l}$ denotes the context embedding of point *x_k_* at the *l*th layer; $h_{k-1}^{i-1}$ and $h_{k+1}^{l-1}$ represent the embeddings of points *x*_*k*−1_, *x_k_* and *x*_*k*+1_ at the previous layer, respectively; and ***s***^*l*−1^ denotes the embedding of the planet node from the preceding layer. LN(·) is the layer normalization function, ReLU(·) is the activation function, and MultiAtt(·) is the multihead attention mechanism.

After all neighbor points **N**(*i*) have been updated, we update the planet node *x_i_* according to the following formula:

$$s^{l}=\mathrm{LN}\left(\text{Re}\mathrm{LU}\left(\text{MultiAtt}\left(s^{l-1},\left[s^{l-1};\;H^{l}\right]\right)\right)\right)$$ (7)

where **H***^l^* represents the embeddings of all the neighbor points at the *l*th layer.

The final feature of the central point *x_i_* in hyperbolic space is obtained through a residual connection, i.e., $f_{i}^{h}=f_{i}^{h}+\lambda \cdot s^{L}$, where λ is a hyperparameter.

Output: After the features $f_{i}^{e}$ and $f_{i}^{h}$ of the central point *x_i_* in Euclidean and hyperbolic space are captured, respectively, we input them into a gate unit module to obtain the output feature $f_{i}^{\prime }$ of the central point *x_i_*:

$$f_{i}^{h^{\prime }}=\log_{O}^{c}\left(f_{i}^{h}\right)={\text{tahn}}^{-1}\left(\sqrt{c}\left\|f_{i}^{h}\right\|\right)\frac{f_{i}^{h}}{\sqrt{c}\left\|f_{i}^{h}\right\|}$$ (8)

$$g=\sigma \left(W\left[f_{i}^{e};\;f_{i}^{h^{\prime }}\right]\right)$$ (9)

$$f_{i}^{\prime }=g\cdot f_{i}^{e}+\left(1-g\right)\cdot f_{i}^{h^{\prime }}$$ (10)

where Eq. 8 transforms the feature from hyperbolic space to Euclidean space, *g* is the gate factor used to control the information contributed from $f_{i}^{e}$ and $f_{i}^{h\prime }$, **W** is the transform matrix, and σ denotes the sigmoid activation function.

#### Graph pooling layer

Similar to [15], we build a hierarchical architecture network by reducing the number of points progressively. The farthest point sampling algorithm [10], operating at a sampling rate of 4, is used to downsample the point clouds. Additionally, the pooling layer is employed to output aggregated features on the coarsened graph that is constructed in correspondence with the sampled points. The feature pooled at each point within the subcloud can be effortlessly extracted by applying a max pooling function to its immediate neighborhood.

#### Optimization

To learn the model parameters and obtain the best segmentation accuracy, we optimize the following loss function:

$$L=\alpha \cdot L_{C}+\beta \cdot L_{\Delta }$$ (11)

where *α*, *β*(*α* + *β* = 1) is a hyperparameter. $L_{C}$ is the cross-entropy loss function, which optimizes the cross-entropy of prediction *p*_*i*,*q*_ and the ground truth *y*_*i*,*q*_, ∀*i* ∈ [1, *n*], *q* ∈ [1, *Q*], to ultimately increase the accuracy of the segmentation task. The triplet loss $L_{\Delta }$ [43] is used to minimize the distance between the anchors and positive samples while maximizing the distance between the anchors and negative samples. The cross-entropy loss $L_{C}$ and triplet loss $L_{\Delta }$ are defined as follows:

$$L_{C}=-\frac{1}{n}\sum_{i=n}^{n}\sum_{q=1}^{Q}y_{i,q}\log\;\left(p_{i,q}\right)$$ (12)

$$L\Delta =\frac{1}{n}\sum_{i=1}^{n}{\left[{\left\|f_{a}-f_{+}\right\|}^{2}-{\left\|f_{a}-f_{-}\right\|}^{2}+\epsilon \right]}_{+}$$ (13)

where ⋅_+_ = max(0, ·), *n* is the number of points, and *Q* is the number of semantic classes. *p*_*i*,*q*_ indicates whether the *i*th point belongs to semantic class *q* or not, whereas *y*_*i*,*q*_ represents the probability of predicting that the *i*th point belongs to semantic class *q*. **f***_a_* denotes the feature vector of the *i*th point, **f**_+_ refers to feature vectors of the points belonging to the same semantic class as the *i*th point, and **f**_−_ represents feature vectors of the points belonging to different semantic classes from the *i*th point. ϵ symbolizes the distance between the anchor points and positive samples.

#### Evaluation criteria

To evaluate the semantic segmentation performance of the SN-MGGE network on the plant point clouds, we calculate 5 quantitative measures, i.e., precision, recall, F1 score, IoU, and overall accuracy (OA) for each semantic class. These measures provide a robust and reliable assessment of the model’s effectiveness from diverse perspectives. Higher values indicate better segmentation. They can be calculated as follows:

$$\mathrm{IoU}=\frac{\mathrm{TP}}{\mathrm{TP}+\mathrm{FP}+\mathrm{FN}}$$ (14)

$$\text{Precision}=\frac{\mathrm{TP}}{\mathrm{TP}+\mathrm{FP}}$$ (15)

$$\text{Recall}=\frac{\mathrm{TP}}{\mathrm{TP}+\mathrm{FN}}$$ (16)

$$F1-\text{score}=2\times \frac{\text{Precision}\times \text{Recall}}{\text{Precision}+\text{Recall}}$$ (17)

$$\mathrm{OA}=\frac{\mathrm{TP}+\mathrm{TN}}{\mathrm{TP}+\mathrm{TN}+\mathrm{FP}+\mathrm{FN}}$$ (18)

where TP, FP, TN, and FN represent the number of true-positive points, false-positive points, true-negative points, and false-negative points in the current semantic class, respectively.

### Phenotype parameter extraction

**Fig. 5.**
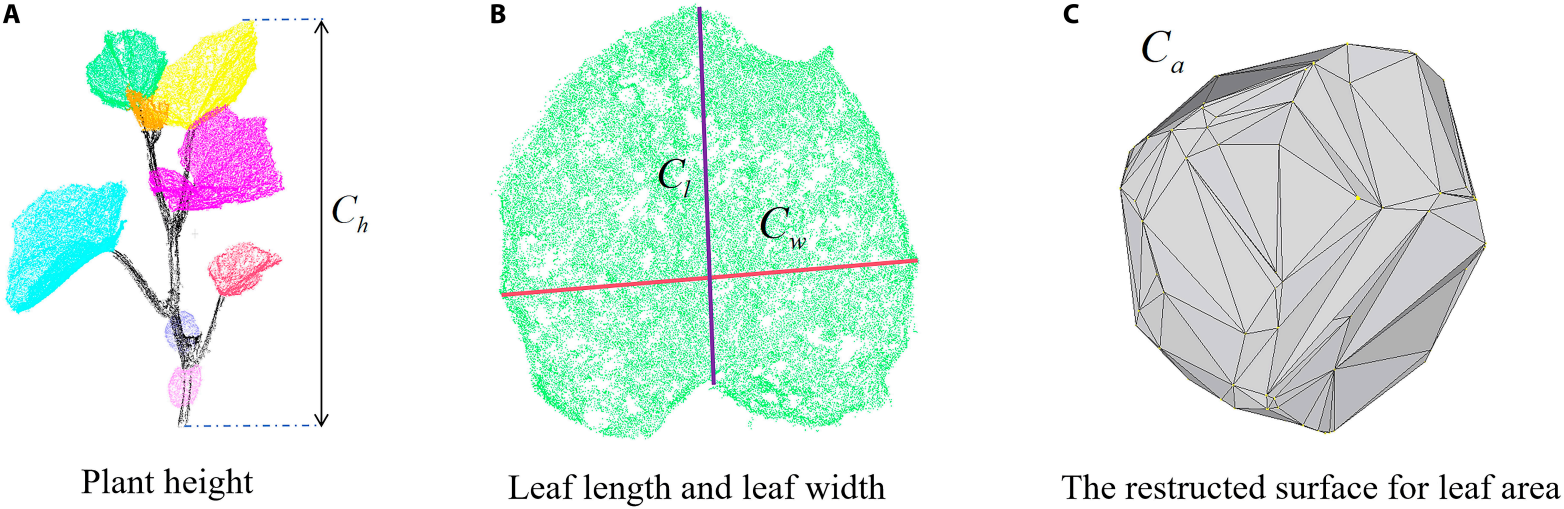
Phenotypic parameters, including (A) plant height *C_h_*, (B) leaf length *C_l_*, leaf width *C_w_*, and (C) leaf area *C_a_*, are extracted.

The leaf point clouds segmented by the SN-MGGE network were employed to extract 4 phenotypic parameters, i.e., plant height, leaf length, leaf width, and leaf area. The plant height *C_h_* was calculated as the difference between the maximum z*_max_* and minimum z_min_ values of the *Z* axis in the point clouds (see Fig. 5A). The leaf length *C_l_* (Fig. 5B, blue) and width *C_w_* (Fig. 5B, red) were measured by calculating the Euclidean distance between the farthest points on the segmented leaves along the *x* and *y* axes. The coordinates of the points within the leaf point clouds were determined via principal components analysis (PCA) [44]. For the leaf area *C_a_*, we used the ball pivoting algorithm (BPA) [45] for estimation, which generates numerous triangular facets by using a 3D sphere with a given radius on the point clouds. The triangle is formed whenever the sphere intersects with 3 points without containing any other point; then, the sphere pivots around an edge (i.e., it revolves around the edge while keeping in contact with the edge endpoints) until it touches another point, forming another triangle. When all the triangles are formed, we calculate the sum of the areas of these triangles to approximate the leaf area (see Fig. 5C).

The formal definitions of plant height *C_h_* and leaf area *C_a_* are as follows:

$$C_{h}=z_{\max}-z_{\min}$$ (19)

$$C_{a}=\sum_{i=1}^{N_{t}}\sqrt{s_{i}\left(s_{i}-e_{i,1}\right)\left(s_{i}-e_{i,2}\right)\left(s_{i}-e_{i,3}\right)}$$ (20)

$$s_{i}=\frac{e_{i,1}+e_{i,2}+e_{i,3}}{2},\forall i\in \left[1,\;N_{t}\right]$$ (21)

where *N_t_* represents the number of triangles. For any triangle *i* ∈ [1, *N_t_*] composed of 3 points (*P*_*i*,1_, *P*_*i*,2_, *P*_*i*,3_), the lengths of the 3 edges are *e*_*i*,1_ = ‖*P*_*i*,1_ − *P*_*i*,1_‖, *e*_*i*,2_ = ‖*P*_*i*,2_ − *P*_*i*,3_‖, and *e*_*i*,3_ = ‖*P*_*i*,3_ − *P*_*i*,1_‖. ‖⋅‖ is the Euclidean distance function.

We employed the coefficient of determination (*R*^2^) and the root mean square error (RMSE) to evaluate the extraction accuracy, which are defined as follows:

$$R^{2}=1-\frac{\sum_{i=1}^{k}{\left(C_{i,g}-{\hat{C}}_{i,g}\right)}^{2}}{\sum_{i=1}^{k}{\left(C_{i,g}-{\bar{C}}_{i,g}\right)}^{2}}$$ (22)

$$\text{RMSE}=\sqrt{\frac{1}{k}\sum_{i=1}^{k}{\left(C_{i,g}-{\hat{C}}_{i,g}\right)}^{2}}$$ (23)

where *k* denotes the number of plants to be compared, and *g* is *h*, *l*, *w*, or *a*. *C*_*i*,*g*_, ${\hat{C}}_{i,g}$, and ${\bar{C}}_{i,g}$ represent the reconstructed, actual, and mean actual values of parameter *g*, respectively. ${\hat{C}}_{i,l}$ and ${\hat{C}}_{i,w}$ were obtained through manual measurement, and the actual leaf area ${\hat{C}}_{i,a}$ was calculated as 0.743 × ${\hat{C}}_{i,l}$ × ${\hat{C}}_{i,w}$.

## Results

### Model training

We implemented the SN-MGGE network on the framework of PyTorch 1.8.2 and used a single NVIDIA GeForce RTX 3090 GPU with 24 GB of RAM to train it. During the training stage, we used the SGD (stochastic gradient descent) optimizer and set the momentum to 0.9 for batch normalization, and the cosine annealing scheme was used to update the learning rate, which ranged from 0.1 to 0.001. The batch size was 4, and the best accuracy result from 200 epochs was used to evaluate the performance of the SN-MGGE network. We set the number *k* of neighborhood sizes to 20 in the KD-Tree-NN algorithm, which was subsequently determined by the results of the ablation experiments.

For the dataset, the number of point cloud samples was expanded from 1,800 to 3,600 via data augmentation, e.g., rotation and uniform downsampling. The dataset was partitioned into a training set, a test set, and a validation set at a ratio of 7:2:1.

**Fig. 6.**
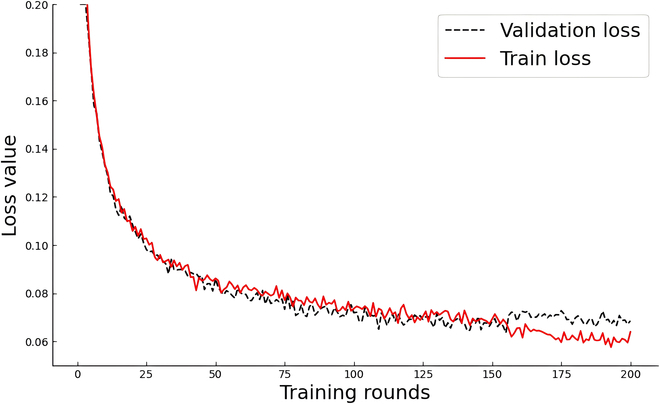
The decreasing trend of loss values during the training and validation stages.

Figure 6 shows the decreasing trend of the loss values. A rapid decrease in the loss values before 50 epochs clearly occurred, suggesting that the parameters were optimized swiftly. Until 150 epochs, the line tended to flatten and eventually stabilized at its minimum value in the validation stage, and they continued to slowly decline in the training stage. To avoid overfitting, training was stopped when the validation loss did not improve within the patience range during multiple consecutive epochs.

### Stem–leaf segmentation results

**Fig. 7.**
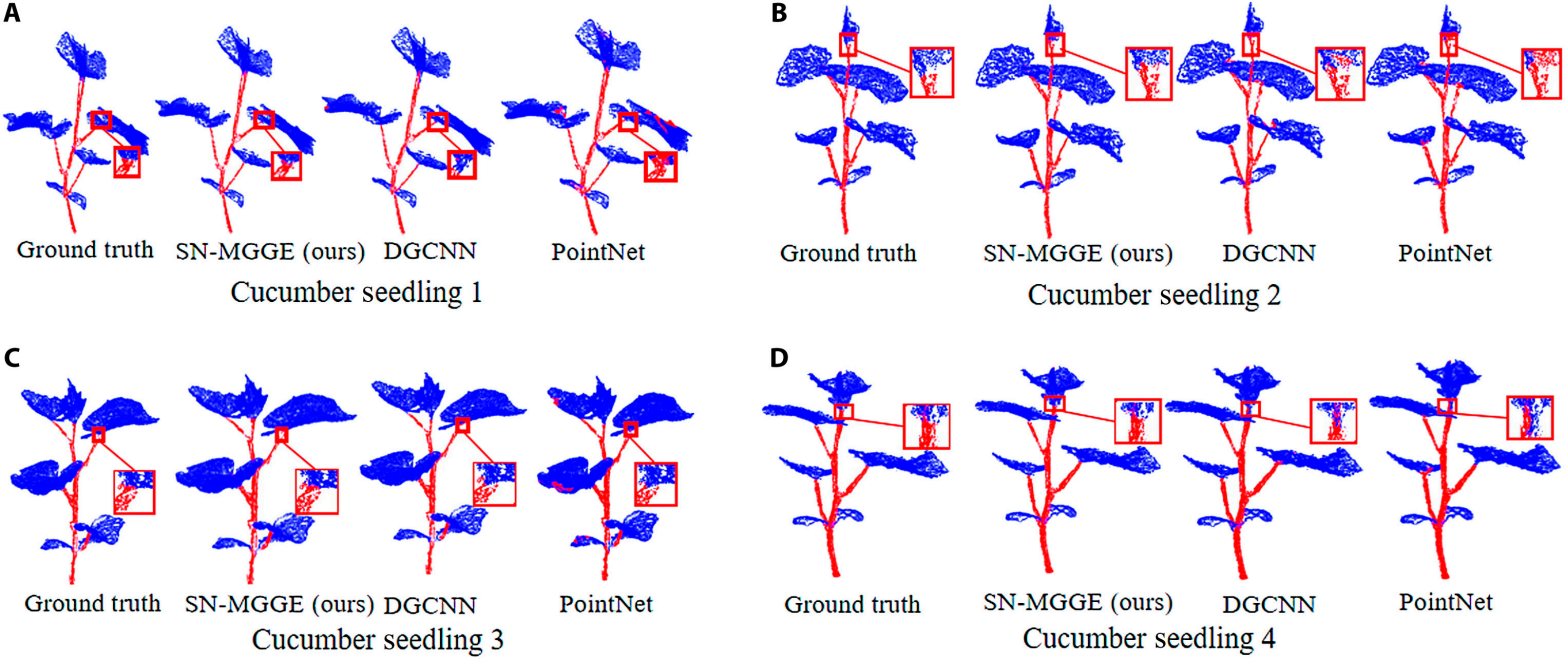
Semantic segmentation results of the SN-MGGE network, DGCNN, PointNet, and ground truth (left) on 4 different cucumber seedlings (A-D); some plant areas are enlarged to provide more details

Figure 7 shows the semantic segmentation results of the SN-MGGE network and the ground truth, PointNet [10], and DGCNN [13]. We can observe that the SN-MGGE network yields excellent segmentation results over stems and leaves by accurately locating the boundary between the stems and leaves compared with DGCNN and PointNet, and only a few false segmentations around the boundary exist (see the red box in Fig. 7). The segmentation outcomes of PointNet and DGCNN were suboptimal, with a notable presence of falsely segmented points observed at the intersections. In addition, we argue that the quality of reconstructed point clouds is the major factor; if high-precision devices such as LiDAR are further used to improve the quality of the point clouds, the quality of the semantic segmentation can also be increased.

To further validate the segmentation performance of the SN-MGGE network, we compared the SN-MGGE network with several mainstream point cloud semantic segmentation networks (i.e., the baseline networks), such as PointNet [10], PointNet++ SSG, PointNet++ MSG [11], DGCNN [13], KPConv [12], and AGConv [15], on the same dataset built in the “Data preprocessing” section, and the parameters of the baseline networks were taken from their original papers. Table 1 shows the comparison results. SN-MGGE achieves the best results, with 94.90% and 97.43% on the performance indicators of the mean IoU (mIoU) and OA, respectively. Compared with PointNet, which does not consider the local neighbor structure, SN-MGGE achieves 12.42% and 7.27% improvements in the mIoU and OA, respectively. Similar to our work, the second-best network (i.e., AGConv) decreases the mIoU and OA by 1.97% and 1.24%, respectively. Unlike AGConv, SN-MGGE fuses the geometric topology structure information on the hyperbolic space channel to enhance the point embeddings in the point clouds to improve the performance on the segmentation task.

**Table 1.** Comparison results of the point cloud segmentation networks

Network	mIoU (%)	OA (%)	Epoch
PointNet [10]	82.48	90.16	200
PointNet2_SSG [11]	86.36	92.52	200
PointNet2_MSG [11]	86.74	92.80	200
DGCNN [13]	90.41	94.32	200
KPConv [12]	91.00	95.53	200
AGConv [15]	92.93	96.19	200
PlantNet [16]	91.16	95.63	200
PSegNet [17]	92.68	95.96	200
SN-MGGE (ours)	94.90	97.43	200

### Instance segmentation results

**Fig. 8.**
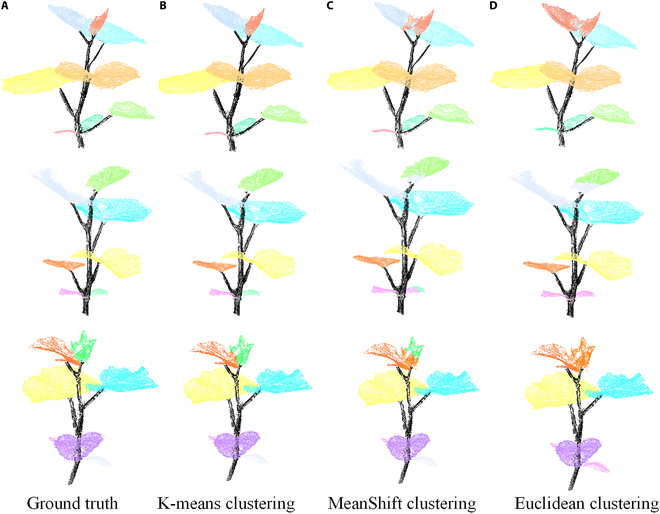
Individual plant segmentation results of the SN-MGGE network with 3 clustering methods. (A) Ground truth. (B) K-means clustering. (C) MeanShift clustering. (D) Euclidean clustering.

On the basis of the above semantic segmentation, the individual leaves were segmented from point clouds containing only leaves through the clustering method. The instance segmentation results are depicted in Fig. 8, where it becomes evident that K-means clustering [39] outperforms MeanShift clustering [46] and Euclidean clustering in terms of accuracy, approaching near-ground truth performance (refer to Fig. 8A and B). In scenarios where leaves exhibit nonoverlapping regions, all 3 clustering methods proficiently segment all leaves, as exemplified by the 3 leaves positioned at the base of the plant in Fig. 8. However, for newly emerged leaves that are tightly clustered (notably at the top of the plant, marked in light red), K-means clustering maintains its superiority, achieving impeccable segmentation. Conversely, Euclidean clustering and MeanShift clustering introduce a significant and modest number of false segmentation points, respectively. We also provide the results of the quantitative analysis of the 3 different clustering methods in Table 2. Specifically, K-means clustering achieves improvements ranging from 2% to 6% across the 4 quantitative measures compared with the other 2 methods.

**Table 2.** Quantitative results of individual plant segmentation across 3 clustering methods

Method	Precision (%)	Recall (%)	F1 score (%)	IoU (%)
K-means	96.18	94.84	95.37	94.11
MeanShift	93.82	92.70	93.28	91.74
Euclidean	90.11	88.36	88.76	87.55

According to the above findings, the K-means clustering method has demonstrated remarkable robustness and precision for segmenting individual leaves within complex point cloud data. In this study, we opted to utilize the K-means clustering method for leaf segmentation. Here, we use the Elbow method [47] to determine the number of clusters and K-means++ [48] to initialize the position of the cluster center point.

### Phenotypic parameter results

**Fig. 9.**
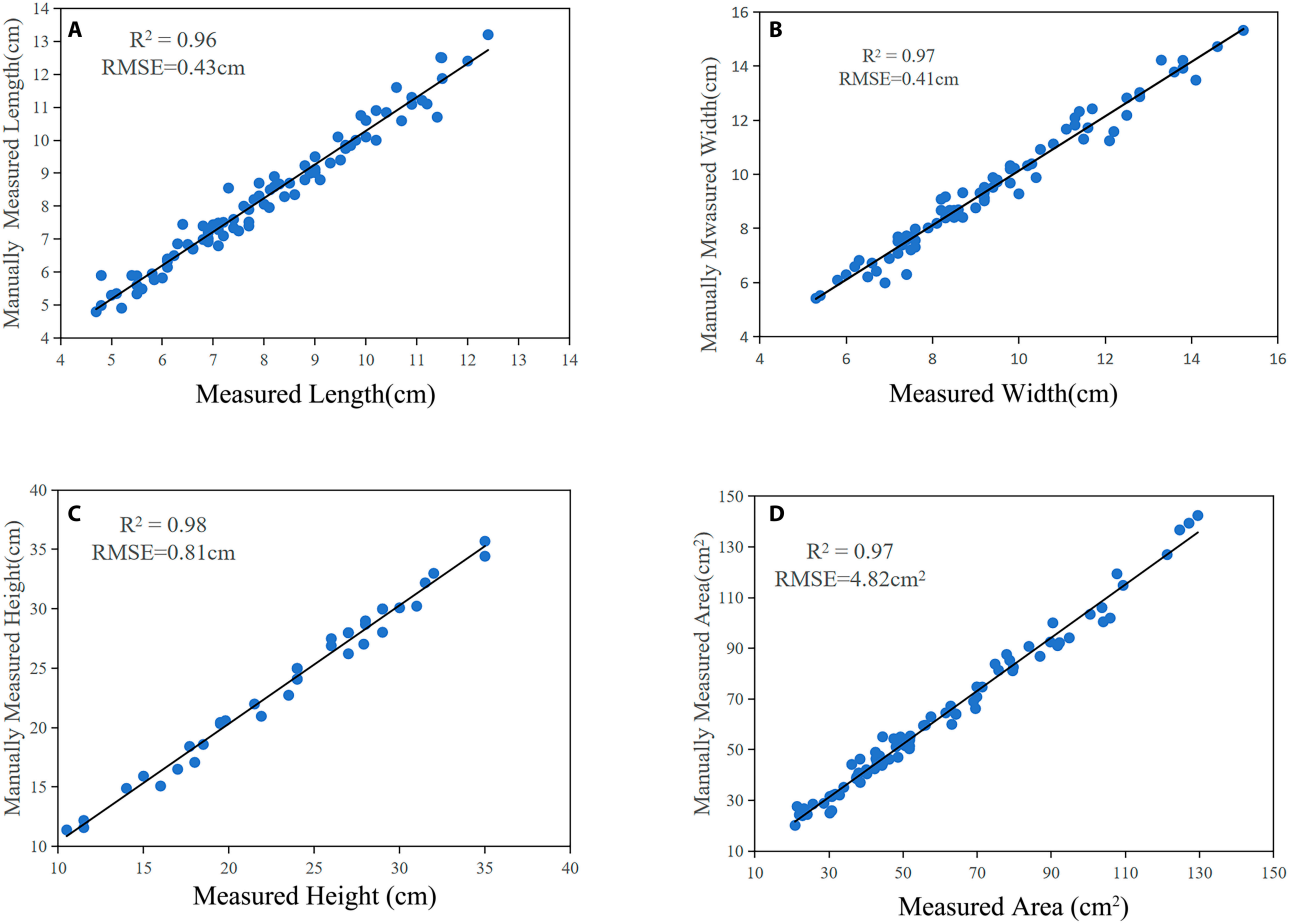
Phenotypic parameter comparison of the extracted values and measured values. (A) Leaf length. (B) Leaf width. (C) Plant height. (D) Leaf area.

We selected 40 cucumber seedlings with approximately 6 to 8 leaves per plant to evaluate the extraction accuracy of 4 phenotypic parameters (i.e., plaint height, leaf length, leaf width, and leaf area). Correlation evaluations of phenotypic parameter estimation versus manual measurements are depicted in Fig. 9. For a total of 40 plants, the *R*^2^ of the estimated plant height was 0.98, which is close to 1, and the RMSE was 0.81 cm (see Fig. 9C).

Nearly 400 segmented leaves were calculated to extract leaf phenotypic parameters, including leaf length, leaf width, and leaf area. As shown in Fig. 9A, B, and D, there was a high correlation between the estimated values and the manually measured values, and the *R*^2^ and RMSE values were 0.96 and 0.43 cm (leaf length), 0.97 and 0.41 cm (leaf width), and 0.97 and 4.82 cm^2^ (leaf area), respectively. Compared with those of plant height, the *R*^2^ values of these 3 phenotypic parameters were relatively low, possibly because the measurement method of calculating the Euclidean distance caused small errors due to uneven leaf surfaces.

In a word, the above experimental results verify the superiority of the proposed segmentation network, although the phenotypic parameters estimated in this study have small errors.

### Ablation study

To further study the impact of each module in the SN-MGGE network, we carry out several ablation experiments on the SN-MGGE network and its variants by removing the relevant module. The detailed experimental results of the SN-MGGE network and 3 variants are shown in Table 3, where “Network A” denotes the SN-MGGE network without the HGS encoder and replaces KD-Tree-NN with KNN, which is consistent with AGConv [15], “Network B” denotes the SN-MGGE network without the HGS encoder, and “Network C” denotes the SN-MGGE network that replaces only KD-Tree-NN with KNN. All the hyperparameters remained the same for the SN-MGGE network and the 3 variants. We observe that the SN-MGGE network outperforms Network B and Network C, which indicates that the KD-Tree-NN and the HGS encoder jointly achieve the best result. In addition, the performance of Network B decreases more than that of Network C, which implies that the HGS encoder can effectively enhance the point embeddings to improve the segmentation accuracy of the SN-MGGE network. Compared with KNN, KD-Tree-NN is better able to locate and retrieve points in a specific area of the point clouds quickly, with improvements of 0.45% and 0.27% in the mIoU and OA, respectively. When KD-Tree-NN was replaced by KNN and the HGS module was removed, i.e., Network A, the SN-MGGE network had the lowest value of 92.99% (96.21%) for the mIoU (OA), with a 1.91% (1.22%) decline.

**Table 3.** Ablation experiment

Network	KD-Tree-NN	HGS	mIoU (%)	OA (%)
Network A	×	×	92.99	96.21
Network B	√	×	93.44	96.48
Network C	×	√	94.82	97.21
Ours	√	√	94.90	97.43

### Impact of neighborhood *K*

In this subsection, we consider the impact of the point neighborhood size (i.e., parameter *K*) on the network performance. Table 4 shows the segmentation results for the performance indicators mIoU and OA for different *K* values. We find that the performance of the SN-MGGE network first rises with an increasing *K* and then decreases. When *K* is set to 20, both the mIoU and OA reach their highest values. If *K* is less than 20, the network may be unable to capture sufficient local information around the center point. However, when *K* is too large (e.g., 30), the network may introduce noise points, such as points that are far from the center and have low correlation, leading to a decrease in the accuracy of the SN-MGGE network.

**Table 4.** Results of the SN-MGGE network for different numbers of neighborhoods

Number of neighborhood (K)	mIoU (%)	OA (%)
5	91.27	94.32
10	93.95	96.58
20	94.90	97.43
30	94.13	97.09

### Robustness test

**Fig. 10.**
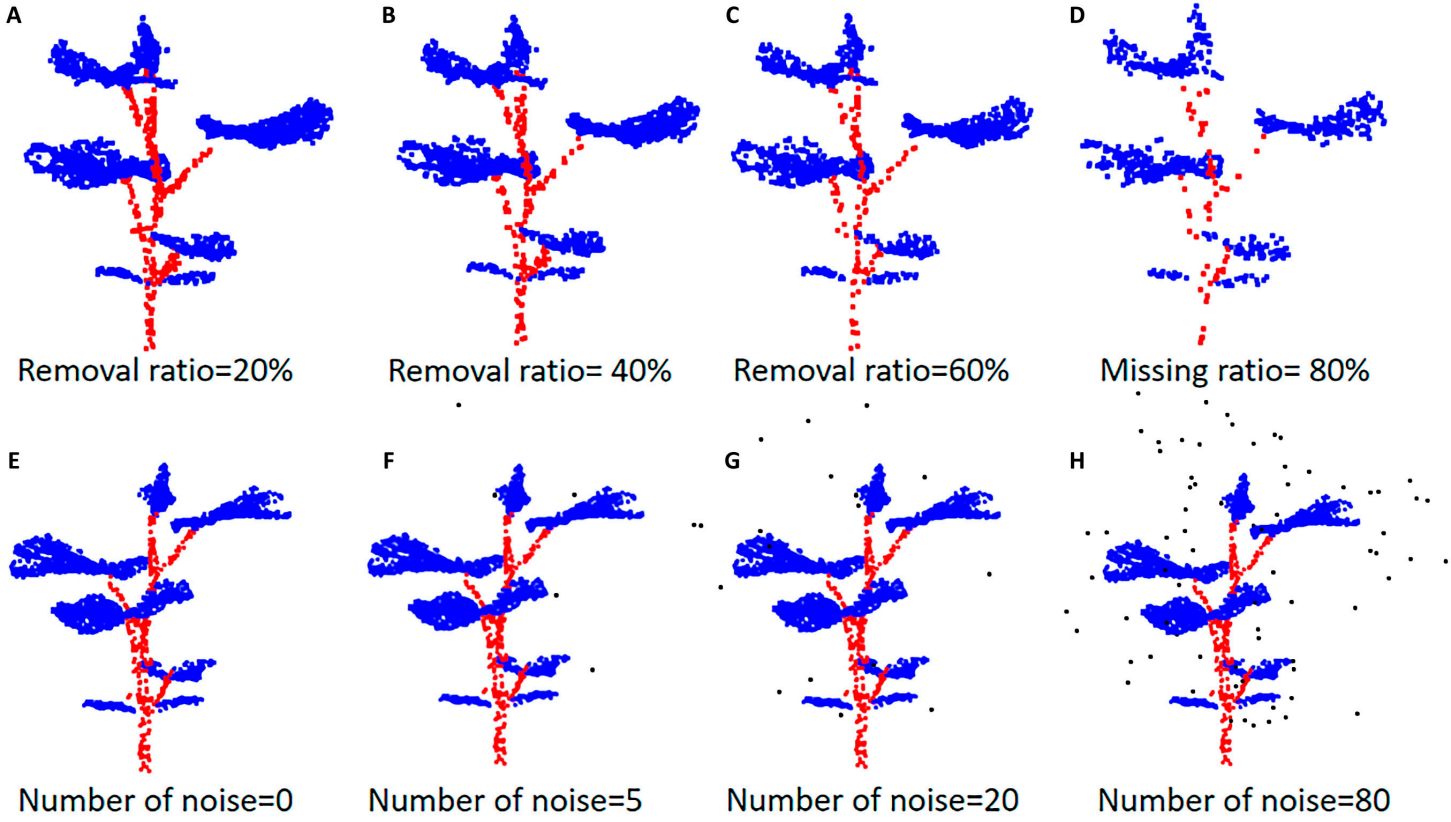
(A to H) Visualization of the plant point clouds with varying removal ratios and numbers of noise.

**Fig. 11.**
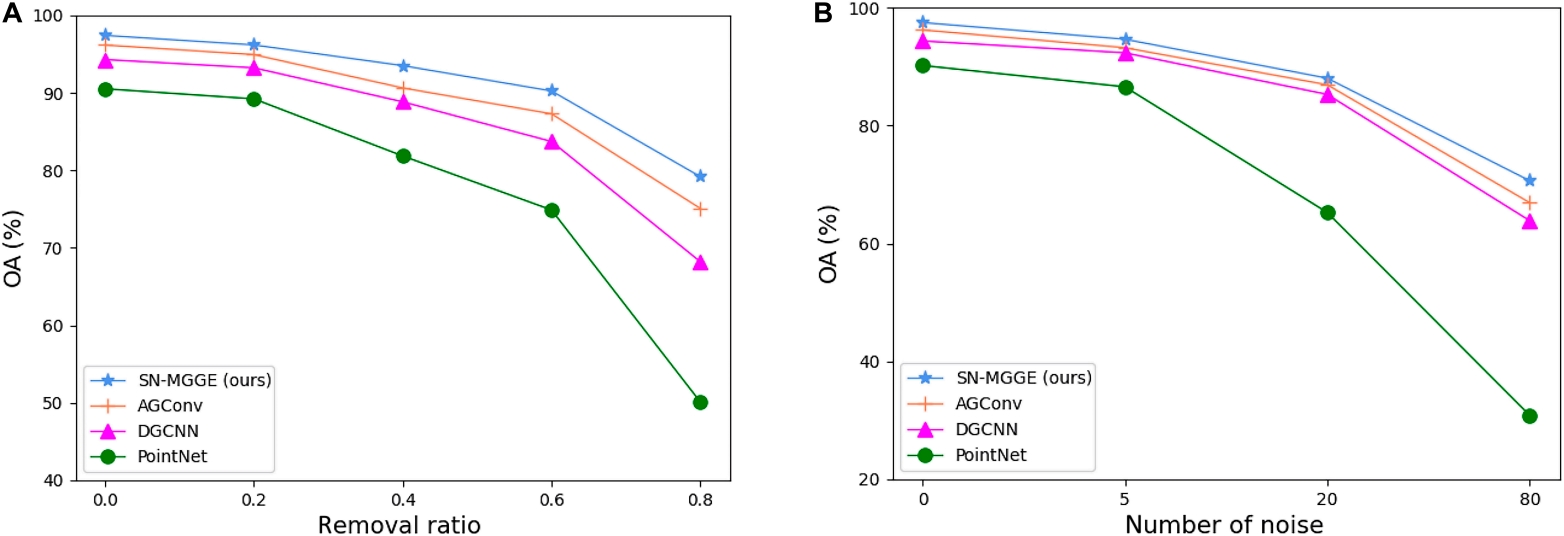
(A) Overall accuracy with different removal ratios. (B) Overall accuracy with different amounts of noise.

On the one hand, we randomly remove 20%, 40%, 60%, and 80% of the points in the point clouds to test our model’s robustness to density. On the other hand, a certain number of selected input points are replaced with uniformly distributed noise points within [−1,1] to test our model’s robustness to noise. We visualize the cucumber seedling models with different removal ratios and numbers of noises in Fig. 10, and the quantitative comparison results are shown in Fig. 11. As shown in Fig. 11A, SN-MGGE can obtain better results than AGConv, PointNet, and DGCNN. Even when the removal ratio is 60%, SN-MGGE can still maintain 90.26% and improve 2.95%, 6.52%, and 15.36% compared with AGConv, DGCNN, and PointNet, respectively. Moreover, SN-MGGE is robust to noise, even in the presence of many noise points (see Fig. 11B). The results of AGConv and DGCNN are very close to those of SN-MGGE; however, PointNet yields significantly poorer results, achieving only a 30.87% accuracy when the number of noise points reaches 80.

In summary, the results demonstrate the suitability of the SN-MGGE network for scenarios involving sparse point cloud data and high levels of noise. This capability is paramount in practical agricultural plant analysis, where factors such as varying lighting conditions and occlusions frequently yield sparse and noisy point clouds captured by devices. Thus, the SN-MGGE network offers a valuable solution for these real-world applications.

### Generalization ability

**Fig. 12.**
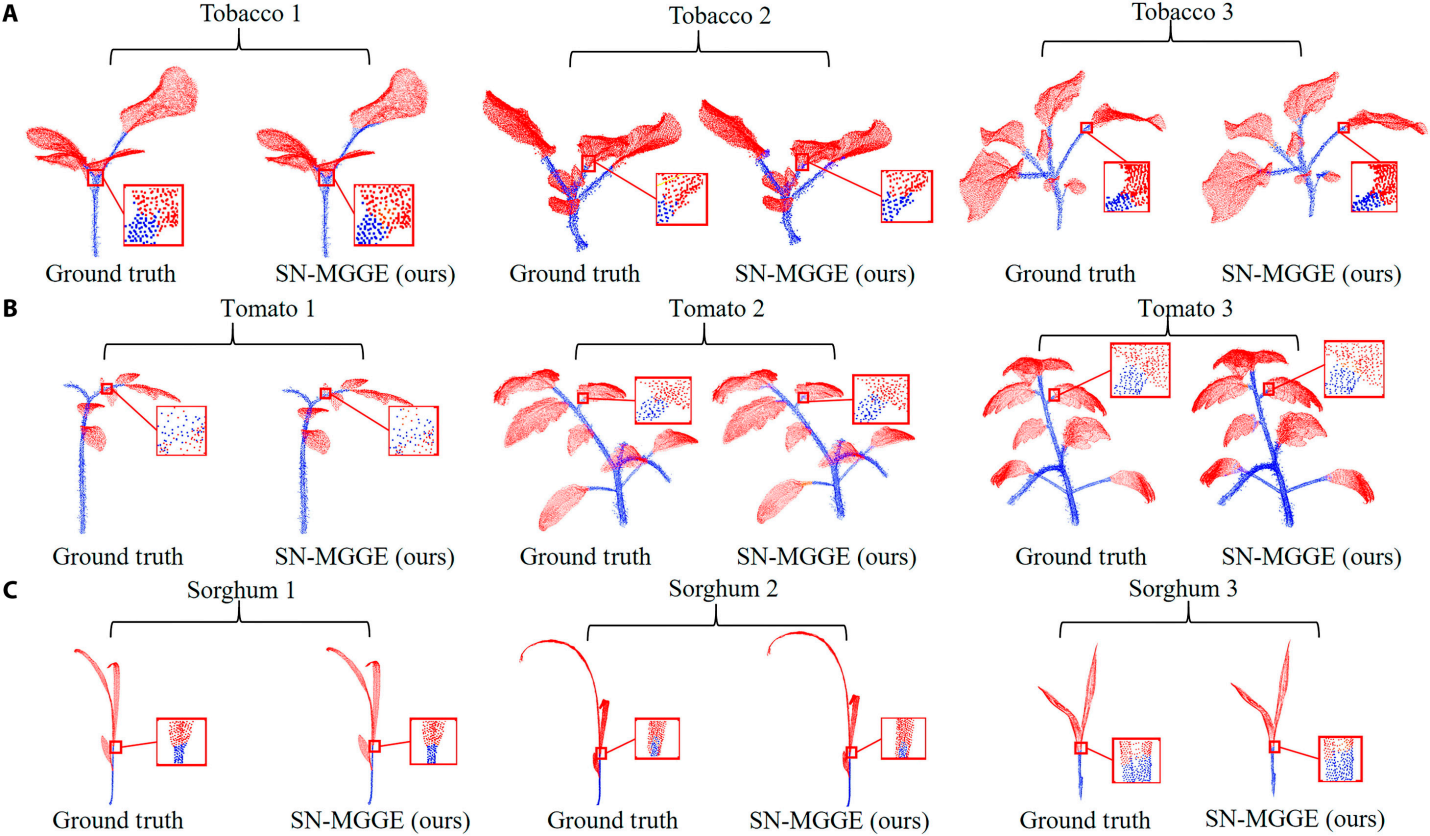
The qualitative instance segmentation comparison of the 3 plant species: (A) tobacco plant, (B) tomato plant, and (C) sorghum plant. Each segmented plant point cloud generated by SN-MGGE is compared with its corresponding ground truth. The enlarged details are within the wireframe.

To comprehensively evaluate the adaptability of the SN-MGGE network to novel datasets, we conducted a comparative analysis with several popular plant semantic segmentation networks, including PlantNet [16] and PSegNet [17], on the plant dataset [49]. The dataset comprises an extensive collection of 546 individual point clouds categorized into 312 tomato point clouds, 105 tobacco point clouds, and 129 sorghum point clouds. Each point cloud within the dataset comprises a substantial range of points, varying from 10,000 to 100,000. We reproduced the network via the parameter configurations recommended in their original paper for comparing networks under the same experimental environment. Table 5 presents a comprehensive quantitative comparison of the 3 networks in terms of their performance on the semantic segmentation task. Notably, the SN-MGGE network is the best performer in most cases and achieves an average improvement of approximately 1% across 4 quantitative measures: precision, recall, F1 score, and IoU. Figure 12 shows the qualitative instance segmentation results of our SN-MGGE network for the 3 plant species. The predicted outputs are correlated with the ground truth annotations. The method accurately segments leaf instances across all 3 leaf types, effectively handling diverse plant morphologies and structures.

**Table 5.** Quantitative comparison of semantic segmentation across the 3 methods

	Method	Tobacco		Tomato		Sorghum		Mean
		Stem	Leaf	Stem	Leaf	Stem	Leaf	
Precision (%)	PlantNet	89.96	96.71	95.53	96.58	89.44	97.27	94.23
	PSegNet	92.82	96.65	96.08	98.09	90.10	97.43	95.20
	SN-MGGE (ours)	93.24	97.78	96.95	98.06	90.68	98.45	95.86
Recall (%)	PlantNet	85.46	92.95	94.89	97.83	85.66	98.21	92.50
	PSegNet	87.40	97.60	95.12	98.29	85.73	98.70	93.81
	SN-MGGE (ours)	90.39	98.94	96.44	98.88	87.92	98.81	95.08
F1 score (%)	PlantNet	86.12	95.01	95.48	97.06	87.44	97.59	93.12
	PSegNet	89.25	97.28	95.29	98.13	87.47	98.06	94.16
	SN-MGGE (ours)	90.74	98.46	96.78	98.56	89.07	98.52	95.36
IoU (%)	PlantNet	79.08	90.10	91.41	94.66	77.95	95.81	88.17
	PSegNet	82.45	94.36	91.73	96.63	78.20	96.22	89.93
	SN-MGGE (ours)	84.62	94.81	92.67	97.10	80.16	96.98	91.06

**Fig. 13.**
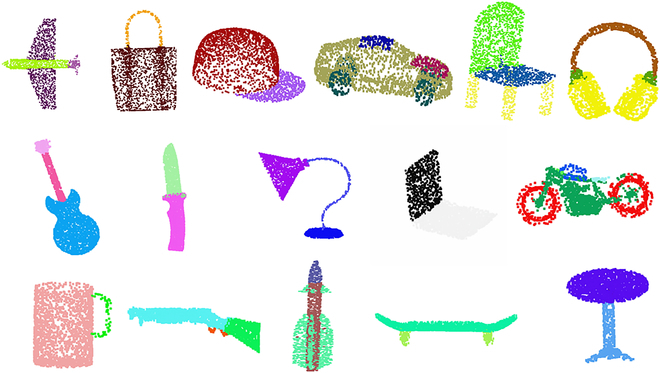
Qualitative results on ShapeNet Part dataset by visualizing the segmentation results from SN-MGGE across all 16 object categories.

Moreover, we also performed an extensive comparative analysis of the SN-MGGE network against several mainstream point cloud segmentation networks, including PointNet [10], PointNet++ [11], DGCNN [13], PointCNN [50], KPConv [12], 3D-GCN [14], AGConv [15], and PointMLP [51], on the widely utilized ShapeNet Part dataset [38]. This dataset comprises 16,881 CAD (computer-aided design) models spanning 16 categories with a total of 50 part labels, where 14,006 models serve for training and 2,874 for testing. Each training model was uniformly sampled to include 2,048 points. The segmentation results, presented in Table 6, reveal that PointNet achieves an accuracy of 83.7%, which lags behind that of the recent AGConv model at 86.4%. This disparity can be attributed to the failure to consider the local (fine-grained) structures surrounding points, namely, their neighborhoods. In contrast, PointNet++ incorporates sampling and grouping modules to learn hierarchical features, resulting in a 1.4% improvement over PointNet. However, it still overlooks the geometric relationships among the points. Models such as DGCNN, PointCNN, KPConv, 3D-GCN, AGConv, and PointMLP achieve accuracies ranging from 85.1% to 86.4%, demonstrating their ability to capture correlations between the points and their neighborhoods. Nevertheless, they do not fully leverage the structural information. Our SN-MGGE network excels, achieving the top score for 9 of 16 object classes and a mean accuracy of 86.8%, surpassing the current state-of-the-art models by approximately 0.4%. The segmentation results for each category, illustrated in Fig. 13, provide qualitative evidence of the effectiveness of our proposed SN-MGGE network. The visualization results vividly showcase the network’s proficiency in accurately segmenting point clouds.

**Table 6.** Comparison of the results of different models for part segmentation on the ShapeNet Part dataset

Method	PointNet	PointNet++	DGCNN	PointCNN	KPConv	3D-GCN	AGConv	PointMLP	SN-GGE
Aero	83.4	82.4	84.0	84.1	84.6	83.1	84.8	83.5	86.2
Bag	78.7	79.0	83.4	86.5	86.3	84.0	81.2	83.4	82.8
Cap	82.5	87.7	86.7	86.0	87.2	86.6	85.7	87.5	91.3
Car	74.9	77.3	77.8	80.8	81.1	77.5	79.7	80.5	78.1
Chair	89.6	90.8	90.6	90.6	91.1	90.3	91.2	90.3	91.5
Ear phone	73.0	71.8	74.7	79.7	77.8	74.1	80.9	78.2	68.2
Guitar	91.5	91.0	91.2	92.3	92.6	90.9	91.9	92.2	91.3
Knife	85.9	85.9	87.5	88.4	88.4	86.4	88.6	88.1	91.1
Lamp	80.8	83.7	82.8	85.3	82.7	83.8	84.8	82.6	87.6
Laptop	95.3	95.3	95.7	96.1	96.2	95.6	96.2	96.2	96.4
Motor bike	65.2	71.6	66.3	77.2	78.1	66.8	70.7	77.5	58.1
Mug	93.0	94.1	94.9	95.3	95.8	94.8	94.9	95.8	93.2
Pistol	81.2	81.3	81.1	84.2	85.4	81.3	82.3	85.4	85.7
Rocket	57.9	58.7	63.5	64.2	69.0	59.6	61.0	64.6	71.7
Skate board	72.8	76.4	74.5	80.0	82.0	75.7	75.9	83.3	79.1
Table	80.6	82.6	82.6	83.0	83.6	82.8	84.2	84.3	85.3
mIoU	83.7	85.1	85.2	86.1	86.4	85.1	86.4	86.1	86.8

## Discussion

The proposed method, which integrates feature extractions of the geometric relationships and shape structures, represents an innovative and valuable approach for plant phenotype analysis. Compared with that of the prevalent segmentation networks, the versatility of the proposed method allows for superior application across various datasets. Nevertheless, certain issues need further improvement in future work.

1. The acquisition and generation of high-quality point clouds is vital for improving the segmentation performance of neural networks and enhancing the accuracy of the plant phenotype extraction results [52]. The constructed point cloud acquisition platform in this study can alleviate interference from environmental factors. First, the use of a white background cloth within the photography studio can effectively eliminate the distractions arising from intricate backgrounds. Moreover, stable lighting conditions are provided by the implementation of LED light sources. Second, we rotate the turntable at low speed while ensuring that there is no wind to capture multiview images because the wind disturbance causes morphological changes in the cucumber seedlings, especially at the leaf tips, and results in a significant error in the extracted plant phenotypic parameters. However, the multiview image acquisition method requires strict environmental control; otherwise, it may result in small deviations during 3D reconstruction, which in turn affects the segmentation accuracy. We consider the direct acquisition of 3D point clouds rather than the image-derived approaches, such as LiDAR and RGB-D.

2. Koma et al. [53] projected the leaf boundary onto a 2D plane perpendicular to the average leaf normal, and then the area of the 2D leaf shape was calculated as the leaf area, which caused many errors in the extracted phenotype parameters, such as leaf width, leaf length, and leaf area. The leaf surface is usually uneven and curly. To this end, we employ the ball-pivoting algorithm (BPA) [45] as a surface reconstruction algorithm, which can quickly and accurately generate smooth 3D surfaces from discrete point cloud data. During the experiment, we iteratively adjusted the radius parameter of the sphere to enable the extracted leaf area to approximate the ground truth value most closely. However, the BPA algorithm has several problems, such as the selection of the ball radius and the creases or valleys on the surface, which can lead to an incomplete surface reconstruction and result in phenotype parameter extraction biases.

## Conclusion

In this study, we propose a novel automatic stem–leaf segmentation network for cucumber seedling point cloud segmentation and phenotypic parameter extraction, namely, SN-MGGE. SN-MGGE achieves high segmentation accuracy by integrating the Euclidean local geometric relationship and the HGS to enhance point embeddings. This method resulted in an mIoU of 94.90% and an OA of 97.43%, significantly improving the performance of semantic segmentation. Furthermore, we evaluated the generalization ability of the SN-MGGE network on 2 datasets, i.e., the plant dataset [49] and the ShapeNet Part dataset [38]. For the plant dataset, the average precision, recall, F1 score, and IoU for stem–leaf segmentation of the 3 plant species reached 95.86%, 95.08%, 95.36%, and 91.06%, respectively. On the ShapeNet Part dataset, SN-MGGE achieves the best result, 86.8%, on the mIoU compared with several popular segmentation networks. On the basis of the segmentation results, 4 plant phenotypic parameters, i.e., plant height, leaf length, leaf width, and leaf area, were accurately measured. The *R*^2^ values of the 4 parameters were all above 0.96. This demonstrated that SN-MGGE meets the requirements for automated and high-precision extraction of plant phenotypic parameters, providing valuable technical support and references for practical agricultural planting. Future works should further the work on SN-MGGE to conduct large-scale plant phenotype analyses on the collected dataset of high-quality point clouds of various plant species other than cucumber seedlings.

## Data Availability

The data and the code are available upon request.
